# DNA repeat arrays in chicken and human genomes and the adaptive evolution of avian genome size

**DOI:** 10.1186/1471-2148-5-12

**Published:** 2005-02-04

**Authors:** Austin L Hughes, Helen Piontkivska

**Affiliations:** 1 Department of Biological Sciences, University of South Carolina, Columbia SC 29208, USA

## Abstract

**Background:**

Birds have smaller average genome sizes than other tetrapod classes, and it has been proposed that a relatively low frequency of repeating DNA is one factor in reduction of avian genome sizes.

**Results:**

DNA repeat arrays in the sequenced portion of the chicken (*Gallus gallus*) autosomes were quantified and compared with those in human autosomes. In the chicken 10.3% of the genome was occupied by DNA repeats, in contrast to 44.9% in human. In the chicken, the percentage of a chromosome occupied by repeats was positively correlated with chromosome length, but even the largest chicken chromosomes had repeat densities much lower than those in human, indicating that avoidance of repeats in the chicken is not confined to minichromosomes. When 294 simple sequence repeat types shared between chicken and human genomes were compared, mean repeat array length and maximum repeat array length were significantly lower in the chicken than in human.

**Conclusions:**

The fact that the chicken simple sequence repeat arrays were consistently smaller than arrays of the same type in human is evidence that the reduction in repeat array length in the chicken has involved numerous independent evolutionary events. This implies that reduction of DNA repeats in birds is the result of adaptive evolution. Reduction of DNA repeats on minichromosomes may be an adaptation to permit chiasma formation and alignment of small chromosomes. However, the fact that repeat array lengths are consistently reduced on the largest chicken chromosomes supports the hypothesis that other selective factors are at work, presumably related to the reduction of cell size and consequent advantages for the energetic demands of flight.

## Background

Genomes sizes (as measured by the DNA mass per diploid nucleus) are smaller on average in birds than in other tetrapod classes, and genome sizes within the class Aves show less variation than those of other tetrapod classes [1,2]. It has been proposed that reduced genome size in birds represents an adaptation to the high rate of oxidative metabolism in birds, which results primarily from the demands of flight [1-4]. Cell size and nuclear genome mass are correlated in vertebrates, and cell sizes of birds are smaller than those of mammals [1]. Smaller cells are advantageous in an animal with a high rate of oxidative metabolism because a smaller cell has a greater surface area per volume of cytoplasm, thus facilitating gas exchange.

An alternative to the hypothesis that the reduced genome size is adaptive is the hypothesis that it resulted from an event of genomic DNA loss that was fixed in the ancestor of all birds due to genetic drift. The fixation of even a deleterious mutation is possible if the population undergoes an extreme bottleneck [5]. Some authors have argued that such a bottleneck may have occurred in the ancestor of birds at the end of the Cretaceous period [6], although this conclusion is not consistent with recent molecular evidence placing the radiation of the avian orders well prior to that time [7].

In order to decide whether genome reduction in birds was adaptive or due to a random event, Hughes and Hughes [8] compared the lengths of corresponding introns of orthologous chicken (*Gallus gallus*) and human (*Homo sapiens*) genes. They found that corresponding introns were significantly shorter in chickens, indicating that numerous independent deletions have occurred in the introns of birds. These results support the hypothesis that genome size reduction in birds is adaptive, since it is unlikely that such a large number of independent deletion events were due to chance alone. Additional evidence in support of the adaptive hypothesis is provided by the observation that a secondary increase in genome size has occurred in avian lineages which have become flightless or have reduced flying ability [9].

It has been suggested that an important factor in genome size reduction in birds has been that birds have lower levels of repetitive DNA than other vertebrates [10]. Genomes of mammals and reptiles are estimated to consist of about 30–50% repeats, while those of birds have been estimated to consist of only 15–20% repeats [10-12]. In birds chromosomes are of two types: a minority of macrochomosomes (3–6 μm in length) and a larger number of microchromosomes (0.5–2.5 μm in length). In the chicken, there are six pairs of macrochromosomes, and thirty-three pairs of microchromosomes [13]. There is a high rate of chiasma formation on avian microchromosomes, and this may be an adaptation that ensures correct pairing of these chromosomes during meiosis and mitosis [14]. Burt [10] proposed that the avoidance of repeats in the avian genome may in turn be an adaptation that enhances the probability of chiasma formation between homologous microchromosomes. This hypothesis and the hypothesis that genome size reduction represents an adaptation to flight are not mutually exclusive, since both factors may be at work simultaneously. Consistent with Burt's hypothesis, Wicker et al. [15] reported that in the chicken genome the ratio of repeats to protein-coding genes is higher on macrochromosomes than on minochromosomes.

The sequencing of a substantial portion of the chicken genome has made it possible to examine quantitatively the distribution of repeating sequences on different chromosomes in the genome. Here we compare the distribution of repeats on 28 sequenced autosomes of chicken with that on the 22 human autosomes in order to test the hypothesis that reduction in repeat density in the avian genome has occurred as a result of adaptive evolution.

## Results

The characteristics of repeat arrays on the 28 sequenced chicken chromosomes are summarized in Table 1. The number of repeat arrays varied from 319 on chromosome 16 to 283,761 on chromosome 1; and the percent of the chromosome occupied by repeats varied from 4.1% on chromosome 32 to 14.9% on chromosome 1. In spite of the considerable variation among chicken chromosomes with respect to the percent of the chromosome occupied by repeats, the overall percentage of the chicken genome occupied by repeats (10.3%) was less than one quarter the percentage of the human genome occupied by repeats (44.9%) (Table 2). Even the most repeat-rich chicken chromosome, chromosome 1, had a repeat density less than one third that of the human genome (Tables 1 and 2). The range of repeat array lengths was much narrower

**Table 1:** DNA sequence repeats on the assembled portion of the chicken genome.

Chromosome	Chromosome length (bp)	No. repeat arrays	Total repeat length (bp) (% of sequence)
1	188,239,860	283,761	27,978,835 (14.9%)
2	147,590,765	214,512	19,430,497 (13.2%)
3	108,638,738	151,571	12,198,434 (11.2%)
4	90,634,903	121,663	8,905,732 (9.8%)
5	56,310,377	69,048	4,638,645 (8.2%)
6	33,893,787	38,873	2,468,824 (7.3%)
7	37,338,262	41,189	2,397,200 (6.4%)
8	30,024,636	33,974	2,086,343 (6.6%)
9	23,409,228	24,255	1,384,475 (5.9%)
10	20,909,726	19,914	1,075,555 (5.1%)
11	19,020054	20,514	1,095,858 (5.8%)
12	19,821,895	19,419	1,116,593 (5.6%)
13	17,279,963	16,894	1,015,160 (5.9%)
14	20,603,938	21,588	1,417,684 (6.9%)
15	12,438,626	11,830	640,595 (5.2%)
16	239,457	319	18,614 (7.8%)
17	10,632,206	9,508	554,602 (5.2%)
18	8,919,268	8,312	574,276 (6.4%)
19	9,463,882	8,635	491,763 (5.2%)
20	13,506,680	12,826	766,482 (5.7%)
21	6,202,554	6,001	359,040 (5.8%)
22	2,228,820	2,636	183,334 (8.2%)
23	5,666,127	4,932	234,823 (5.7%)
24	5,910,111	5,435	356,373 (6.0%)
26	4,255,270	3,385	188,003 (4.4%)
27	2,668,888	2,833	252,335 (9.5%)
28	4,731,479	5,183	446,256 (9.4%)
32	1,018,878	806	42,242 (4.1%)

**Table 2:** Features of DNA sequence repeats on human and chicken autosomes.

	Human	Chicken
No. chromosomes analyzed	22	28
Total sequence length (bp)	2,864,255,932	901,598,378
No. repeat arrays	4,698,717	1,160,319
Total repeat length (bp) (% of sequence)	1,287,381,310 (44.9%)	92,440,122 (10.3%)
Mean repeat array length (bp) [median] (range)	274.0 [188.0] (7–160,603)	79.7 [25.0] (6–7,096)
Mean no. repeat arrays per chromosome [median] (range)	213,578 [219,247] (57,109–409,783)	41,440 [14,860] (319–283,761)

**Figure 1:**
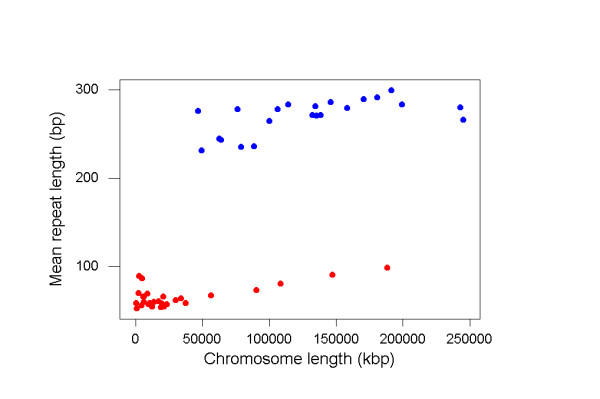
The percentage of the chromosome occupied by repeats (% repeats) as a function of chromosome length in human (*blue dots*) and chicken (*red dots*).

In the chicken genome, there was a significant positive correlation (r = 0.847; P < 0.001) between chromosome length and the percentage of the chromosome occupied by repeats (% repeats)(Figure 1). The four largest chicken chromosomes (chromosomes 1–4) contributed strongly to this positive correlation. In the case of the four largest chromosomes, there was a clear linear relationship between chromosome length and % repeats (Figure 1). In the human genome, there was also a positive, but non-significant correlation (r = 0.412; n.s.) between chromosome length and % repeats (Figure 1).

As illustrated in Figure 1, % repeats values for human chromosomes were considerably higher than those for chicken chromosomes, even when the chromosome length were similar. An analysis of covariance was used to compare % repeats between the two species, with chromosome length as a covariate. There was a significant difference between species (P < 0.001) and a significant effect of chromosome length (P < 0.001), but there was not a significant interaction between species and chromosome length. These results imply that there is a similar slope to the linear relationship between chromosome length and % repeats in the two species, but that the % repeats values for human are significantly higher than those for chicken.

Comparison of summary statistics human and chicken genomes showed that both mean and median repeat array lengths were considerably greater in the former species than in the latter (Table 2). In order to provide a statistical test of this difference that was not biased by the presence of different array types in the two genomes, we conducted paired tests on the 294 simple sequence repeat types shared by the two genomes (Table 3). Both the mean array length and the maximum array length were significantly greater in human than in chicken (Table 3). By contrast, the minimum array length did not differ significantly between chicken and human (Table 3). In order to test whether these differences between the two species were due mainly to the influence of the smaller chicken chromosomes, we repeated the analysis using only the four largest chicken chromosomes (chromosomes 1–4). In this case also, both the mean array length and the maximum array length were again significantly greater in human than in chicken, while the minimum array length was not significantly different between species (Table 3).

**Table 3:** Mean (± S.E.) of variables describing simple sequence repeat types shared between human and chicken.

	Human	Chicken	P (paired-sample t-test)
All chicken chromosomes (294 repeat types):			
Mean array length (bp)	83.6 ± 2.8	58.1 ± 1.5	< 0.001
Minimum array length (bp)	22.1 ± 1.6	22.1 ± 0.7	n.s.
Maximum array length (bp)	457.9 ± 25.0	193.5 ± 8.1	< 0.001
			
Chicken chromosomes 1–4 (286 repeat types):			
Mean array length (bp)	83.8 ± 2.9	56.4 ± 1.7	< 0.001
Minimum array length (bp)	21.7 ± 1.6	24.8 ± 1.1	n.s.
Maximum array length (bp)	466.7 ± 25.5	153.3 ± 6.9	< 0.001

## Discussion

Tabulation of DNA repeat arrays in the assembled portion of the chicken autosomes showed the overall percentage of repeats to be 10.3%. This value is similar to, but slightly lower than, previously published estimates (about 15%) based on reassociation kinetics [11,12]. By contrast, a similar tabulation in the human autosomes showed the overall percentage of repeats to be 44.9%. Because the value for chicken is substantially lower than the mammalian value, the results support the hypothesis that a relative scarcity of repeating DNA is a major factor in causing the relatively compact size of the avian genome [15].

Moreover, when simple sequence repeat array types shared between chicken and human genomes were compared, mean repeat array length and maximum repeat array length were significantly lower in the chicken than in human. The fact that these differences occurred consistently in nearly 300 distinct array types is evidence that the reduction in repeat arrays in the chicken has involved numerous independent evolutionary events. Mutational changes to simple sequence repeat arrays typically involve slippage events that either decrease or increase the number of repeat units [16]. The fact that simple sequence repeat arrays are shorter in the chicken than in the human implies that mutational events increasing array length have been eliminated by selection in the chicken to a greater extent than in human. Such long arrays might have included some that were inherited from the ancestors of birds and others that arose due to mutational events within Aves. In either case, the evidence for numerous, independent events of elimination of long arrays implies that reduction of DNA repeat length and thus of overall genome size in birds has occurred as a result of adaptive evolution.

There were substantial differences among chicken chromosomes with respect to the percentage of the chromosome occupied by repeats, and % repeats increased significantly as a function of chromosome length. This trend implies that the avian genome is characterized by an especially pronounced avoidance of longer repeats on the smaller chromosomes. This finding is consistent with the hypothesis of Burt [10] that the reduction of repeating DNA in avian genomes is adaptive in permitting chiasma formation and alignment of microchromosomes.

However, even the largest chicken chromosomes had repeat densities much lower than human chromosomes of similar length (Figure 1). This implies that avoidance of repeats on microchromosomes cannot be the sole factor at work in repeat avoidance in avian genomes. This interpretation is further supported by the fact that mean repeat array length and maximum repeat array length of repeat types shared between chicken and human genomes were significantly lower on the largest four chicken chromosomes than in human. Thus, the largest chicken chromosomes, like the rest of the chicken genome, showed a pattern indicating adaptive reduction of repeat array length. Our results imply that some other selective factor besides the need for alignment of minichromosomes contributes to genome size reduction in birds. Together with previous evidence [9], the results are thus consistent with the hypothesis that genome size reduction in birds is adaptive in that it leads to reduction of cell size and thus is advantageous in view of the energetic demands of flight.

## Methods

The chicken (*Gallus gallus*) genome assembly (May 2004 freeze, release 25.1b.1) was downloaded from Ensembl web site at . Only autosomes were used in the analyses; data were available for chromosomes 1 through 24, 26, 27, 28 and 32. We extracted Ensembl annotations of the features of repeat arrays (including repeat name, start and end positions on the chromosome, and orientation). The human genome assembly (May 2004 freeze, build 35 (hg17)) was downloaded via the UCSC Genome Browser . Repeat information based on the RepeatMasker annotations (repeat name, start and end positions on the chromosome and orientation) was extracted from the UCSC genome annotation database. Only autosomes (chromosomes 1 through 22) were used. For both chicken and human, repeats tallied included simple sequence repeats, class I elements, class II elements, low-complexity regions, and satellite regions. In addition, we compared between genomes a set of 294 simple sequence repeats which were present in both genomes; i.e., repeats of the same short nucleotide motif were present in both genomes. For these 294 repeat types, mean, minimum and maximum length of repeat arrays were compared in pairwise fashion between human and chicken.

## Authors' contributions

HP gathered and summarized the data. ALH conducted statistical analyses and wrote the manuscript. Both authors read and approved the final manuscript.

## References

[B1] Szarski H (1976). Cell size and nuclear DNA content in vertebrates. Int Rev Cytol.

[B2] Tiersch TT, Wachtel SS (1991). On the evolution of genome size in birds. J Hered.

[B3] Szarski H (1983). Cell size and the concept of wasteful and frugal evolutionary strategies. J Theor Biol.

[B4] Wachtel SS, Tiersch TR (1993). Variations in genome mass. Comp Biochem Physiol B.

[B5] Ota T, Nei M (1995). Evolution of immunoglobulin VH pseudogenes in chickens. Mol Biol Evol.

[B6] Alvarez LW (1983). Experimental evidence that an asteroid impact led to the extinction of many species 65 million years ago. Proc Natl Acad Sci USA.

[B7] Kumar S, Hedges SB (1998). A molecular timescale for vertebrate evolution. Nature.

[B8] Hughes AL, Hughes MK (1995). Small genomes for better fliers. Nature.

[B9] Hughes AL (1999). Adaptive Evolution of Genes and Genomes.

[B10] Burt DW (2002). Origin and evolution of avian minichromosomes. Cytogenet Genome Res.

[B11] Epplen JT, Leipoldt M, Engel W, Schmidtke J (1978). DNA sequence organization in avian genomes. Chromosoma.

[B12] Schmid M, Nanda L, Guttenbach M, Steinlein C, Hoehn M, Schartl M, Haaf T, Weigend S, Fries R, Buerstedde JM, Wimmers K, Burt DW, Smith J, A'Hara S, Law A, Griffin DK, Bunstead N, Kaufman J, Thomson PA, Burke T, Groenen MA, Crooijmans RP, Vignal A, Fillon V, Morisson M, Pitel F, Tixier-Boichard M, Ladjali-Mohammedi K, Hillel J, Maki-Tanila A, Cheng HH, Delany ME, Burnside J, Mizuno S (2000). First report on chicken genes and chromosomes 2000. Cytogenetics and Cell Genetics.

[B13] Smith J, Bruley CK, Paton IR, Dunn I, Jones CT, Windsor D, Morrice DR, Law AS, Masabanda J, Sazanov A, Waddington D, Fries R, Burt DW (2000). Differences in gene density on chicken macrochromosomes and microchromosomes. Animal Genet.

[B14] Rodionov AV, Chelysheva LA, Solovei IV, Myakoshina YA (1992). Chiasmata distribution on lampbrush chromosomes of the chicken *Gallus gallus domesticus*: recombination hotspots and their possible significance for correct disjunction of homologous chromosomes in the first meiotic division. Genetica.

[B15] Wicker T, Robertson JS, Schulze SR, Feltus FA, Magrini V, Morrison JA, Mardis ER, Wilson RK, Peterson DG, Paterson AH, Ivarie R (2005). The repetitive landscape of the chicken genome. Genome Res.

[B16] Schlötterer C (2000). Evolutionary dynamics of microsatellite DNA. Chromosoma.

